# Effects of (de)motivating supervision styles on junior doctors’ intrinsic motivation through basic psychological need frustration and satisfaction: an experimental vignette study

**DOI:** 10.1007/s10459-024-10344-0

**Published:** 2024-06-25

**Authors:** Wieke E. van der Goot, Nico W. Van Yperen, Casper J. Albers, A. Debbie C. Jaarsma, Robbert J. Duvivier

**Affiliations:** 1 Martini Academy, Martini Hospital, Groningen, The Netherlands; 2 University of Groningen, University Medical Center Groningen, Lifelong Learning, Education and Assessment Research Network (LEARN), Groningen, The Netherlands; 3 Department of Psychology, University of Groningen, Groningen, The Netherlands; 4 Faculty of Veterinary Medicine, University of Utrecht, Utrecht, The Netherlands; 5 Parnassia Psychiatric Institute, The Hague, The Netherlands

**Keywords:** Basic psychological needs, Intrinsic motivation, Self-determination theory, Supervision style, Vignette methodology

## Abstract

In clinical practice, junior doctors regularly receive supervision from consultants. Drawing on Basic Psychological Needs Theory, consultants’ supervision styles are likely to affect junior doctors’ intrinsic motivation differently in terms of psychological need frustration and psychological need satisfaction. To examine the effects of (de)motivating supervision styles, we conducted two experimental vignette studies among junior doctors. In Study 1 (*N* = 150, 73.3% female), we used a 2 (need support: *high* vs. *low*) x 2 (directiveness: *high* vs. *low*) *between*-subjects design and, in Study 2, a *within*-subjects design with the same factors (*N* = 46, 71.7% female). Both studies revealed a consistent positive effect of need-supportive supervision styles on psychological need satisfaction (+), need frustration (-), and intrinsic motivation (+). Particularly in Study 2, the main effect of need-supportive styles was strengthened by supervisor’s directiveness. Moreover, in both studies, the effects of supervision styles on intrinsic motivation were explained through psychological need frustration and psychological need satisfaction. We discuss the implications of these findings for postgraduate clinical training.

**Supplementary Information:**

The online version contains supplementary material available at 10.1007/s10459-024-10344-0.

Junior doctors’ intrinsic motivation for clinical practice may be influenced by several factors. For example, the supervision style of their consultant affects junior doctors’ basic psychological needs for autonomy, competence, and relatedness (Deci & Ryan, 2008; Ryan & Deci, 2000b). This may result in frustration or satisfaction of these needs, and in turn can lead to jeopardised or enhanced intrinsic motivation. A loss of intrinsic motivation may subsequently lead to health problems and maladaptive work behaviour, whereas increased intrinsic motivation may lead to better well-being and more job satisfaction (Vansteenkiste & Ryan, 2013). Hence, to effectively guide and support junior doctors’ development and functioning, consultants need to adapt their supervision styles to junior doctors’ psychological needs (Kilminster et al., 2007; Kilminster & Jolly, 2000). To better align and train effective styles of supervision, we first need to understand the effects of (de)motivating supervision styles on junior doctors’ intrinsic motivation. Drawing on Basic Psychological Needs Theory (e.g., Deci & Ryan, 2000; Vansteenkiste et al., 2010), we examined if and how consultants’ supervision styles may affect junior doctors’ intrinsic motivation differently through psychological need frustration and psychological need satisfaction.

## Basic psychological needs theory

**Fig. 1:**
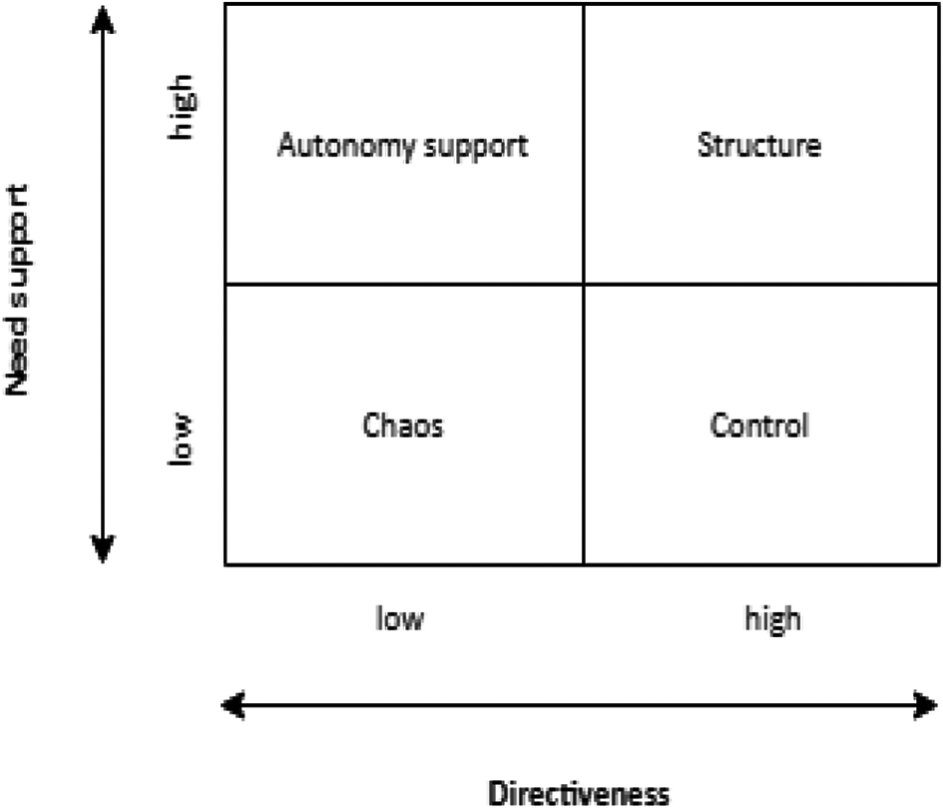
Framework for conceptualising supervision styles that differ in need support and directiveness

Basic Psychological Needs Theory is one of the six mini-theories of Self-Determination Theory (SDT, Ryan & Deci, 2017; Vansteenkiste et al., 2010), with each mini-theory incorporating different aspects of the socio-contextual conditions that hamper or facilitate well-being, flourishing, and healthy development (Ryan & Deci, 2017). Basic Psychological Needs Theory posits that support of the basic psychological needs of autonomy, competence, and relatedness is essential for individuals’ well-being, growth, and development (Deci & Ryan, 2000, 2008; Ryan, 1995). The need for autonomy refers to a desire to be able to act volitionally, with a sense of choice and freedom (DeCharms, 1968 cf., Deci & Ryan, 2008; Ten Cate et al., 2011). The need for competence refers to a desire to feel effective, to have a feeling of ability, sufficiency, or success (White, 1959 cf., Elliot et al., 2017; Ten Cate et al., 2011). The need for relatedness refers to a desire to feel connected and have a sense of belongingness in relation to others and in communities (Baumeister & Leary, 1995; Ten Cate et al., 2011). An increasing body of literature indicates that need frustration and need satisfaction are two different concepts and lead to different detrimental and beneficial outcomes, respectively (Vansteenkiste & Ryan, 2013; Vansteenkiste et al., 2020). Note that a lack of need satisfaction does not imply that needs are frustrated. Conversely, absence of need frustration does not necessarily mean that needs are satisfied. Therefore, both frustration and satisfaction of psychological needs are relevant because each may uniquely predict intrinsic motivation, and subsequently, have a different effect on well-being, performance, and professional development (Haerens et al., 2018; Ryan & Deci, 2000a; Vansteenkiste & Ryan, 2013). To examine if and how consultants’ supervision styles may affect junior doctors’ intrinsic motivation, we relied on a framework (see Fig. 1) in which four (de)motivating styles were distinguished on the basis of two dimensions: Need Support and Directiveness, which are discussed next.

## Need-supportive supervision

*Low* need-supportive supervision styles may be harsh, demanding, and critical (i.e., a controlling style) or passive, absent, and conflicting (i.e., a chaotic style). These styles may undermine or frustrate junior doctors’ needs for autonomy, competence, and relatedness (Vansteenkiste & Ryan, 2013). In contrast, *high* need-supportive supervision styles are characterised by an understanding, encouraging, and non-judgmental approach (i.e., an autonomy-supportive or structuring style). These styles may foster junior doctors’ needs for autonomy, competence, and relatedness (Vansteenkiste & Ryan, 2013). Indeed, an increasing body of literature shows that need-supportive styles are negatively related to psychological need frustration, and positively associated with psychological need satisfaction in education (Aelterman et al., 2018; Vansteenkiste et al., 2019), sports (Bartholomew et al., 2011; Delrue et al., 2019), and work (Hardré & Reeve, 2009; Van den Broeck et al., 2010), including nursing practice (Duprez et al., 2019) and medical education (Neufeld & Malin, 2020). Hence, in *Hypothesis 1*, we pose that *compared with high need support, low need support leads to more psychological need frustration and less psychological need satisfaction*.

## Directive supervision

Directive supervision styles are characterised by structure, clear expectations, and guidelines for behaviour (i.e., structuring and controlling styles). Absence of direction is characterised by little structure, unclear expectations, and few guidelines for behaviour (i.e., autonomy-supportive and chaotic styles; Jang et al., 2010). Supervision styles that are *low* in need support and *high* in directiveness (i.e., controlling styles) may be perceived as especially controlling because these styles actively thwart basic psychological needs by micro-managing, thereby suppressing volitional functioning (Vansteenkiste et al., 2010). Supervision styles that are *low* in need support and *low* in directiveness (i.e., chaotic styles) may be perceived as need depriving. While psychological needs are not supported, this style does not necessarily jeopardise volitional functioning and leaves room to find need support elsewhere (Vansteenkiste et al., 2010). Therefore, *in Hypothesis 2*, we pose that *compared with high need support, low need support leads to more psychological need frustration and less psychological need satisfaction, particularly in the case of high directive supervision.*

## Explaining intrinsic motivation through basic psychological needs

Central to Basic Psychological Needs Theory is the proposition that supporting (and not thwarting) individuals’ basic psychological needs is a prerequisite for intrinsic motivation. The underlying principle is that frustration (versus satisfaction) of psychological basic needs jeopardises (versus facilitates) internalisation and integration of relevant social norms and values, which hampers (versus stimulates) responsible, self-determined behaviour (Deci & Ryan, 2000, 2008; Ten Cate et al., 2011; Vansteenkiste & Ryan, 2013). In other words, support of all three basic psychological needs is essential for the internalisation and integration of the norms and values of clinical practice. This is needed for learning and healthy development as it helps junior doctors to self-regulate (future) behaviour and experience it as self-determined (i.e., originating from and aligned with yourself). Thus, frustration of the three psychological needs undermines intrinsic motivation (Bartholomew et al., 2011; Costa et al., 2015; Ryan & Deci, 2000a), whereas satisfaction of the three psychological needs promotes intrinsic motivation (Deci & Ryan, 2000, 2008; Vansteenkiste et al., 2010). However, the mediation of psychological need frustration and psychological need satisfaction has not been sufficiently investigated in Health Professions Education (HPE, Kusurkar et al., 2011; Orsini et al., 2016). To fill this gap, in *Hypothesis 3a*, we pose that *compared with high need support, low need support leads to less intrinsic motivation through more psychological need frustration and less psychological need satisfaction*. Similarly, in *Hypothesis 3b*, we pose *that compared with high need support, low need support leads to less intrinsic motivation through more psychological need frustration and less psychological need satisfaction, particularly in the case of high directive supervision.*

## The present research

**Fig. 2:**
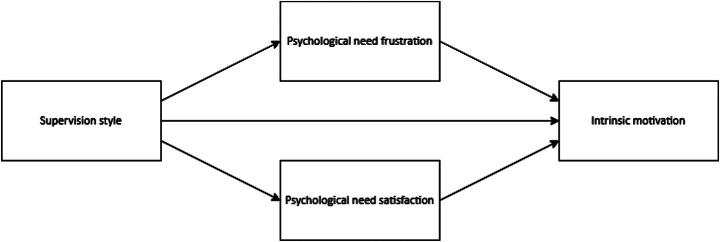
Research model

In this research, we conducted two studies to examine the effects of four different supervision styles (see Fig. 1) on intrinsic motivation through psychological need frustration and psychological need satisfaction (see Fig. 2). To test our hypotheses, we adopted an experimental vignette methodology with written scenarios. This methodology allows (1) testing of causal relationships, (2) controlling of the independent variables (i.e., supervision styles) in different experimental conditions, and (3) assessment of their effects without the ethical constraints that may arise when these are investigated in practice (Aguinis & Bradley, 2014; Atzmüller & Steiner, 2010). In Study 1, we relied on a *between*-subjects design to compare the effects of different supervision styles on our dependent variables. In contrast, in Study 2, we relied on a *within*-subjects design to compare the effects of different supervision styles on the same participants: that is, each participant compared and evaluated all four styles (Aguinis & Bradley, 2014; Atzmüller & Steiner, 2010). In combining these two designs, we utilised the strengths of each approach. *Between*-subjects designs are more suitable for testing the effectiveness of experimental manipulations and do not suffer from comparison effects because participants are randomly assigned to only one of the experimental conditions. In *within*-subjects designs, however, the comparisons between experimental conditions may result in findings that are better generalisable to clinical practice. That is, in clinical practice, junior doctors are likely to experience different supervision styles in different situations and contexts.

## Study 1

### Method

#### Design

**Scenario development and pilot testing.** The scenarios were developed in 2019 and consisted of short, written descriptions of situations that junior doctors may encounter in clinical practice. We used written scenarios to effectively reflect authentic clinical situations (external validity), and included manipulated supervision styles as the intervention (internal validity). To optimise the external validity of both scenarios and supervision styles, we had four junior doctors evaluate the preliminary scenarios and provide feedback to make the scenarios more realistic and recognisable.

The experimental conditions (i.e., manipulations) addressed the behavioural responses of a supervisor (i.e., supervision styles). These responses were based on four different (de)motivating styles adapted from Aelterman et al. (2018). The *high* need-supportive, *low* directive style (*autonomy support*) was characterised by an attitude of understanding (e.g., the supervisor asks for junior doctors’ opinions on how to deal with this case). The *high* need-supportive, *high* directive style (*structure*) was characterised by an attitude of guidance (e.g., the supervisor provides tips on how the junior doctors can improve their performance and says that s/he trusts that they will improve). The *low* need-supportive, *low* directive style (*chaos*) was characterised by chaos (e.g., the supervisor accepts everything the junior doctors say and creates uncertainty by not providing any guidance at all). The *low* need-supportive, *high* directive style (*control*) was characterised by an attitude of pressure (e.g., the supervisor points out that poor performance is not acceptable and that the junior doctors should stick to the rules and guidelines). Finally, we developed and pilot-tested manipulation checks to test if the participants could differentiate between our four supervision styles (see Fig. 1). Moreover, these manipulation checks served as a prerequisite to test if the manipulation of the experimental conditions was effective in Study 1. Supplementary Information 1 (SI1) shows the pilot study, scenarios, and supervision styles.

**Study 1.** In Study 1, we used a full factorial 2 (need support: *high* vs. *low*) x 2 (directiveness: *high* vs. *low*) *between*-subjects design. We used four vignettes presenting scenarios of hypothetical situations at emergency departments that participants may encounter when they are on-call. Each scenario included a patient who was presented to the participant. We instructed the participants to imagine that they would do the initial assessment and/or clinical examination of this patient and next call their supervisor for help or advice. We used four different vignettes to improve the ecological validity of Study 1, because situations in clinical practice are diverse. By using four vignettes, we allowed the participants to form their judgments of the supervision styles based on four clinical situations.

#### Procedure & participants

Participants were recruited from 15 teaching hospitals and two university medical centres in the Netherlands between May and September 2021. Junior doctors who worked or had experience working in an emergency department qualified for participation. An invitation e-mail contained information about the study. Participants could then access the study via a hyperlink. Participation was voluntary. A digital informed consent form preceded the survey. The survey software Qualtrics randomly assigned each participant to one of the four experimental conditions. First, participants answered questions on socio-demographic information. Next, participants read four scenarios presenting one of the four supervision styles (i.e., the experimental condition). After reading the scenarios, the participants rated their psychological need frustration, psychological need satisfaction, intrinsic motivation, and, finally, the manipulation checks. All measures were assessed in Dutch. Participants received no compensation for their time. Ethical approval was obtained from the Netherlands Association for Medical Education (NVMO, file #2020.7.1).

We received 254 submissions, of which 154 (60.6%) were complete. Incomplete submissions were removed from the dataset.^1^ Testing for outliers in the manipulation checks revealed 19 participants with extreme scores (SD >|3|); only three of these participants showed strongly deviating response patterns in the manipulation check items. Therefore, these three participants were excluded from further analysis. In addition, one submission showed a multivariate outlier pattern of the six dependent variables (i.e., Mahalanobis distance (df = 6) > 22.46); this participant was also excluded from further analysis. The final sample comprised of 150 junior doctors (*n* = 110, 73.3% female^2^), who worked as junior doctors not-in-training (*n* = 53, 35.3%) or as Post-Graduate Medical Education (PGME) trainees (*n* = 97, 64.7%). More specifically, 49 junior doctors (32.7%) were in their first or second PGME year, 31 junior doctors (20.7%) were in their third or fourth PGME year, and 19 junior doctors (12.7%) were in their fifth or sixth PGME year. Participants’ ages ranged from 25 to 42 years (*M* = 29.81, *SD* = 3.06), they worked in 22 different specialities, and trainees were being trained in 20 different PGME programmes. In the final sample, the *high* need support, *low* directiveness condition (autonomy support) consisted of 32 participants; the *high* need support, *high* directiveness condition (structure) consisted of 44 participants; the *low* need support, *low* directiveness condition (chaos) consisted of 39 participants; and the *low* need support, *high* directiveness condition (control) consisted of 35 participants.

#### Measures

**Manipulation checks.** Our previously pilot-tested instrument that measured need support and directiveness (three items per factor) was used to check whether the manipulation of the experimental conditions (i.e., need-supportive versus directive supervision styles) worked. Participants rated these six items on a seven-point Likert scale ranging from 1 (*not at all*) to 7 (*completely*). Example items are, “In the four scenarios, the supervisor…” (a) “… attunes to my questions” (need support, α = 0.97), and (b) “… gives direction” (directiveness, α = 0.93).

**Basic Psychological Need Frustration and Satisfaction.** Participants’ work-related autonomy, competence, and relatedness frustration and satisfaction were measured using the 24-item *Basic Psychological Need Satisfaction and Frustration* scale (four items per subscale), developed by Chen et al. (2015). Items were rated on a five-point Likert scale ranging from 1 (*not true at all*) to 5 (*completely true*). The item scores of each subscale were averaged to calculate reliability scores (Cronbach’s α). We adapted the general stem to the specific context of our study: “By the way in which the supervisor reacts…”. Items were only adapted on a minor level to ensure a correct sentence structure following the general stem.

**Intrinsic motivation.** Participants’ intrinsic motivation was measured using a three-item version of the enjoyment scale (Carpenter et al., 1993; Van Yperen, 1998). The general stem was, “If you were on call with the supervisor from the scenarios, would you…”. The three items were (1) “… *enjoy* doing your work?”, (2) “… have *fun* doing your work?”, and (3) “… *like* your work?”. The three items were followed by a five-point Likert scale ranging from 1 (*not at all)* to 5 (*very much)*.

#### Statistical analyses

We used SPSS (IBM Corp, 2019; version 26) for descriptive analyses, manipulation checks (ANOVA), and the 2 × 2 MANOVA. To test whether the effects of supervision styles (X) on intrinsic motivation (Y) were mediated through psychological need frustration and satisfaction (M_1 − 6_), we used the structural equation modelling (SEM) package lavaan (Rosseel, 2012; version 0.6–12) in *R* (R Core Team, 2022; version 4.2.1).

In our SEM models, the independent variable (X), i.e., the experimental condition, was a constant, set to 1. As a result, the coefficient from X to M (i.e., psychological need frustration and satisfaction, *a-*path) was equivalent to the average score of M in experimental condition X. The coefficient between M and Y (*b-*path) could be modelled. The coefficients of both paths were multiplied to give ‘*ab*’, which was our parameter of the indirect effect of X on Y, through M. When this indirect effect was significant, we concluded that M significantly mediated the effect of X on Y.

To test for the unique predictive effects of all our mediating variables (M_1_– M_6_), we built parallel mediation models. These models partitioned the effect of X on Y through six indirect effects (‘*ab*’ coefficients for each M) and the remaining direct effect (*c’*). In all our mediation models, we bootstrapped our analyses 1,000 times to estimate robust values of our standard errors (*SE*), 95% confidence intervals (95% CI), and Sobel’s statistic (Sobel, 1982).

## Results

Table 1 shows the means, standard deviations, and correlations of all variables. Intrinsic motivation correlated negatively with need frustration (1–3) and positively with need satisfaction (4–6). Age, sex, and training level showed no meaningful relationship with the variables of our research model (see Fig. 2).

**Table 1:** Cronbach’s Alpha’s, means, standard deviations, and correlations of the variables (Study 1)

Variables	Cronbach’s α	*M*_*Study 1*_	*SD*_*Study 1*_	2	3	4	5	6	7
1. Autonomy frustration	.87	2.17	0.98	.72	.68	−.53	−.69	−.52	−.68
2. Competence frustration	.94	2.15	1.15	–	.73	−.53	−.71	−.53	−.69
3. Relatedness frustration	.93	1.91	1.06		–	−.54	−.70	−.61	−.69
4. Autonomy satisfaction	.86	3.20	0.90			–	.75	.63	.68
5. Competence satisfaction	.96	3.51	1.14				–	.69	.80
6. Relatedness satisfaction	.93	3.09	0.91					–	.62
7. Intrinsic motivation	.96	3.50	1.19						–

*Notes. N*_Study 1_ = 150. Correlations observed are significant at the *p* <.001 level

### Manipulation checks

To test whether the experimental manipulation of need support was successful, we performed a 2 (need support: *high* versus *low*) x 2 (directiveness: *high* versus *low*) analysis of variance (ANOVA). As expected, the need-support manipulation check revealed a strong main effect of need support, *F*(1, 146) = 473.91, *p* <.001, ${\eta }_{p}^{2}$ =.76. Participants in the *high* need-support conditions perceived more need support than participants in the *low* need-support conditions. This indicates that the experimental manipulation of need support was successful.

A similar 2 × 2 ANOVA on the directiveness manipulation check revealed the expected strong main effect of directiveness, *F*(1, 146) = 136.83, *p* <.001, ${\eta }_{p}^{2}$ =.48. Participants in the *high* directiveness conditions (*M* = 5.12, *SD* = 1.03) perceived more directiveness than participants in the *low* directiveness conditions (*M* = 3.01, *SD* = 1.38). Hence, we concluded that the experimental manipulation of directiveness was successful, too.

### Hypothesis testing

In *Hypothesis* 1, we posited that *compared with high need support, low need support leads to more psychological need frustration and less psychological need satisfaction*. To test this hypothesis, we conducted a 2 (need support: *high* versus *low*) x 2 (directiveness: *high* versus *low*) MANOVA with all seven dependent variables to test the effects of the experimental conditions on psychological need frustration and psychological need satisfaction. As shown in Table 2, the results indicate a strong multivariate main effect of need support. Compared with the *high* need-support conditions, participants in the *low* need-support conditions reported significantly more (*ps* <.001) need frustration and less (*ps* <.001) need satisfaction (see Table SI2). These results provide empirical support for *Hypothesis 1*.

**Table 2:** 2 × 2 MANOVA results with need support (high versus low) and directiveness (high versus low) as between-subject factors (study 1)

	Multivariate *F*(7, 140)	V			Univariate *F*(1, 146)	$\eta _p^2$
Need support	36.39***	.65	1.	Autonomy frustration	116.77***	.44
			2.	Competence frustration	106.03***	.42
			3.	Relatedness frustration	107.97***	.43
			4.	Autonomy satisfaction	75.26***	.34
			5.	Competence satisfaction	186.62***	.56
			6.	Relatedness satisfaction	94.33***	.39
			7.	Intrinsic motivation	167.20***	.53
Directiveness	2.71*	.12	1.	Autonomy frustration	2.67	.02
			2.	Competence frustration	2.45	.02
			3.	Relatedness frustration	0.04	.00
			4.	Autonomy satisfaction	5.32*	.04
			5.	Competence satisfaction	3.98*	.03
			6.	Relatedness satisfaction	0.36	.002
			7.	Intrinsic motivation	1.10	.01
Need support x Directiveness	2.39*	.11	1.	Autonomy frustration	0.30	.002
			2.	Competence frustration	7.02**	.05
			3.	Relatedness frustration	3.06	.02
			4.	Autonomy satisfaction	0.27	.002
			5.	Competence satisfaction	0.17	.001
			6.	Relatedness satisfaction	0.93	.01
			7.	Intrinsic motivation	0.16	.001

*Note* *p <.05. **p <.01. ***p <.001. V = Pillai’s Trace (because Box’s M was significant)

**Fig. 3:**
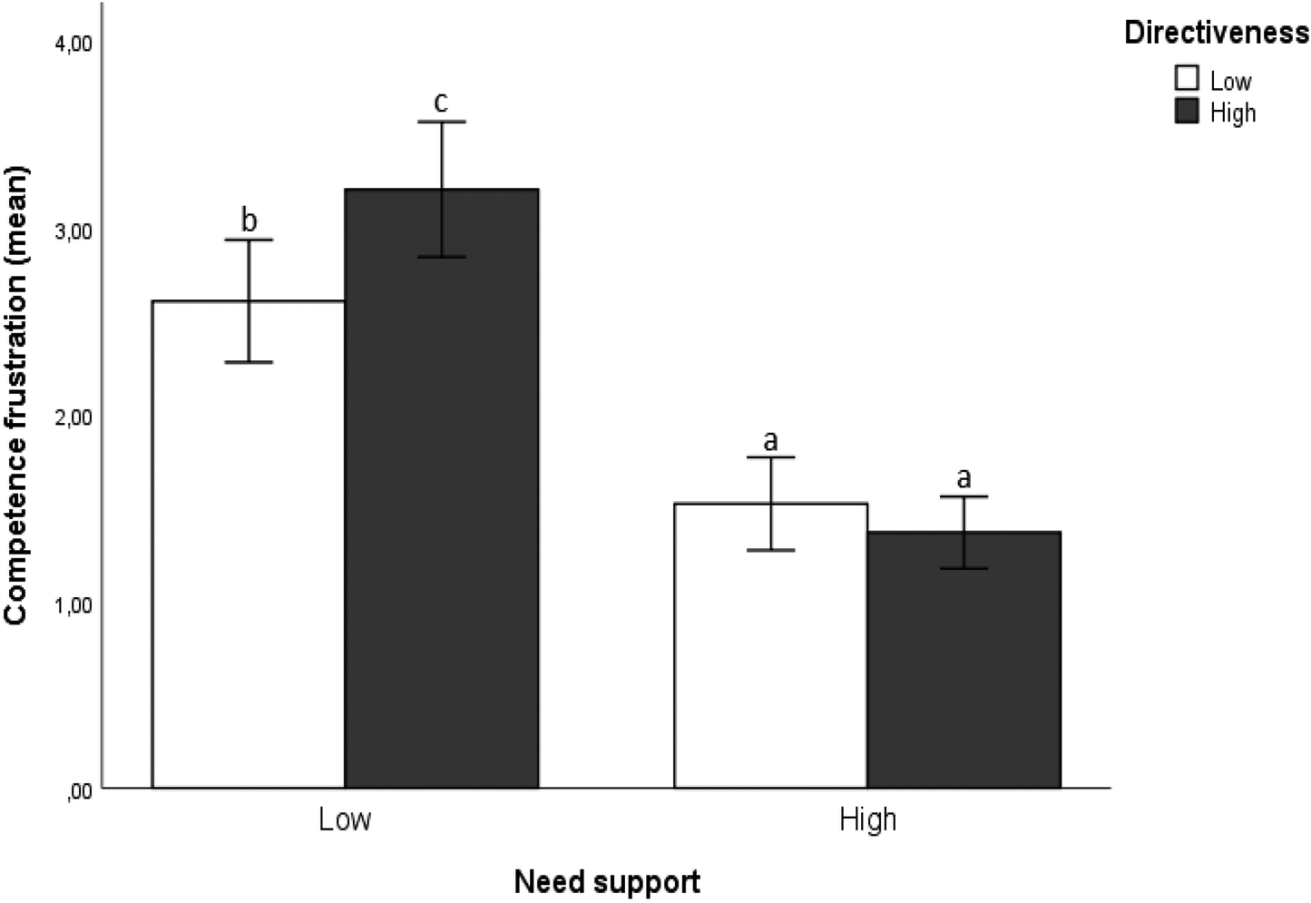
High directive supervision strengthened the negative effects of low need-supportive supervision styles on competence frustration (study 1). *Note*. This figure illustrates the univariate results of the significant multivariate interaction effect of need support and directiveness for competence frustration. Different letters signal differences of *p* <.05 at the minimum. *M*_*Low NS, Low DIR*_ = 2.61, *SD*_*Low NS, Low DIR*_ = 1.01; *M*_*Low NS, High DIR*_ = 3.21, *SD*_*Low NS, High DIR*_ = 1.05; *M*_*High NS, Low DIR*_ = 1.52, *SD*_*High NS, Low DIR*_ = 0.69; and *M*_*High NS, High DIR*_ = 1.37, *SD*_*High NS, High DIR*_ = 0.63

In *Hypothesis 2*, we posited that *compared with high need support, low need support results in more psychological need frustration and less psychological need satisfaction, particularly in the case of high directiveness.* However, the expected multivariate interaction effect between need support and directiveness was observed for competence frustration only (see Table 2). Figure 3 shows that, compared with participants in the *high* need-support conditions, participants in the *low* need-support conditions reported significantly more competence frustration, particularly when directiveness was *high*. Hence, *Hypothesis 2* was empirically supported, but for competence frustration only.

**Fig. 4:**
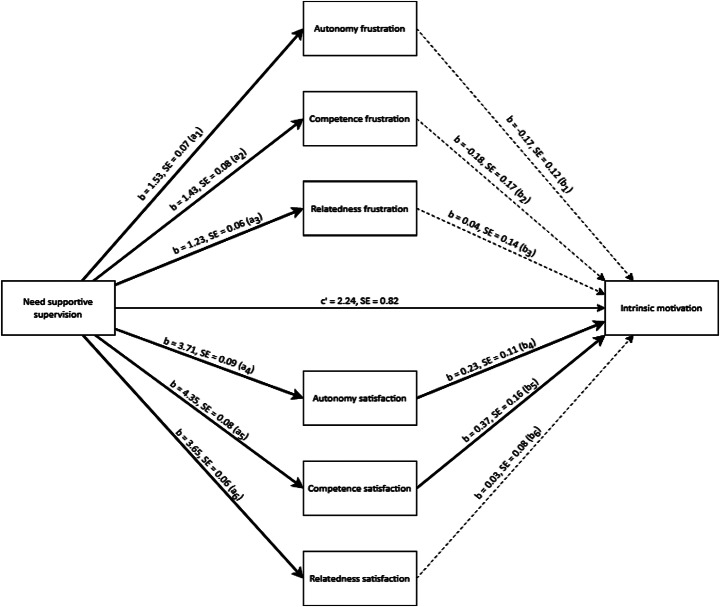
Parallel mediation model of basic psychological need frustration and need satisfaction for high need-supportive supervision styles and intrinsic motivation (study 1). *Note*. The solid lines are significant effects with *p* <.05

In *Hypothesis 3a*, we posited *that compared with high need support, low need support leads to less intrinsic motivation through more psychological need frustration and less psychological need satisfaction*. As shown in Table 2, we found a multivariate main effect of need support on intrinsic motivation. Compared with participants in the *high* need-support conditions, participants in the *low* need-support conditions reported significantly less intrinsic motivation. Table 3 shows that the effect of *high* need support on intrinsic motivation was negatively mediated through autonomy and competence frustration, and positively mediated through autonomy, competence, and relatedness satisfaction. The effect of *low* need support on intrinsic motivation was negatively mediated through autonomy, competence, and relatedness frustration, and positively mediated through autonomy, competence, and relatedness satisfaction. Next, we tested the unique predictive values of both need frustration and need satisfaction on intrinsic motivation in a parallel mediation model. Figure 4 shows that when all mediating variables were considered, autonomy satisfaction and competence satisfaction uniquely and significantly predicted intrinsic motivation only in the *high* need-support conditions (see Table SI3). Hence, we concluded that the effects of supervision styles on intrinsic motivation can be explained significantly through both need frustration and need satisfaction, but the strongest predictors of intrinsic motivation were autonomy satisfaction and competence satisfaction. Thus, we found empirical support for *Hypothesis 3a*.

**Table 3:** Indirect effects of high and low need-supportive supervision styles on intrinsic motivation through psychological need frustration and psychological need satisfaction (study 1)

	High need support						Low need support					
Indirect effects	*ab*	*SE*	95% CI		*p*	Sobel	*ab*	*SE*	95% CI		*p*	Sobel
			*LL*	*UL*					*LL*	*UL*		
Separate models for each variable*												
1. Autonomy frustration	-0.72	0.28	-1.25	-0.19	.009	-0.16	-1.10	0.36	-1.86	-0.45	.002	-0.42
2. Competence frustration	-0.78	0.27	-1.24	-0.26	.005	-0.18	-0.98	0.29	-1.56	-0.42	<.001	-0.37
3. Relatedness frustration	-0.52	0.32	-1.21	0.03	.110	-0.12	-1.06	0.27	-1.60	-0.55	<.001	-0.40
4. Autonomy satisfaction	1.81	0.52	0.74	2.81	<.001	0.42	1.45	0.30	0.85	2.01	<.001	0.55
5. Competence satisfaction	2.93	0.66	1.39	4.02	<.001	0.67	1.48	0.27	0.96	2.05	<.001	0.56
6. Relatedness satisfaction	0.99	0.34	0.35	1.71	.004	0.23	1.12	0.39	0.30	1.86	.004	0.43
Summarised models for need frustration and need satisfaction**												
Need frustration	-0.96	0.32	-1.48	-0.33	.003	-0.22	-1.61	0.37	-2.36	-0.92	<.001	-0.61
Need satisfaction	2.85	0.70	1.37	4.06	<.001	0.65	2.02	0.40	1.22	2.86	<.001	0.77

*Note* *p <.008 is considered significant (Bonferroni correction with a factor 6 due to multiple model testing for both satisfaction and frustration). **p <.05 is considered significant. Furthermore, the bootstrapped 95% Confidence Intervals (95% CI) of the indirect effects are considered significant when the parameters of the Lower Limit (*LL*) and Upper Limit (*UL*) do not include zero. All indirect effects (*ab*) are unstandardized. Both standard error (*SE*) and 95% CI, and Sobel’s statistic ((*ab*) / (*ab* + *c’*)), are 1000 bootstrapped estimates. Direct effects are not presented in this table, because the direct effect (*c’*) is the value of the total effect (*c*), which is the mean value of intrinsic motivation for *high* versus *low* need-supportive styles, minus the indirect effect (*ab*)

In *Hypothesis 3b*, we posited that *compared with high need support, low need support leads to less intrinsic motivation through more need frustration and less need satisfaction, particularly in the case of high directiveness*. As shown in Table 2, we found no multivariate interaction effect on intrinsic motivation. Thus, we found no empirical support for *Hypothesis 3b*.

## Study 2

Study 1 provided strong evidence that need-supportive supervision styles had a positive effect on psychological need satisfaction (+), psychological need frustration (-), and intrinsic motivation (+). Furthermore, especially autonomy satisfaction and competence satisfaction positively predicted intrinsic motivation in the *high* need-support condition. Unexpectedly, we did not find evidence for the additional detrimental effect of *low* need-supportive, *high* directive supervision styles. A possible explanation is that the detrimental effects of this style become more salient when participants are aware of alternative supervision styles. In addition, comparing different supervision styles may feel more realistic since junior doctors will typically deal with different supervision styles in daily practice. Hence, in Study 2, we aimed to conceptually replicate the findings of Study 1 in a *within-*subjects design (Aguinis & Bradley, 2014; Atzmüller & Steiner, 2010). That is, participants evaluated and compared all four supervision styles (in random order).

### Method

#### Design

In Study 2, we relied on a full factorial 2 (need supportive supervision: *high* versus *low*) x 2 (directive supervision: *high* versus *low*) *within*-subjects design. In this study, we instructed participants to evaluate all four supervision styles in the context of one particular scenario (i.e., Scenario 2 from Study 1). This scenario was deemed most suitable for the relatively inexperienced junior doctors that participated in Study 2. They typically ask more frequently for, and consequently, tend to receive more, supervision from a consultant.

#### Procedure & participants

Participants were informed about the study and accessed it via a hyperlink, and followed a procedure similar to that described in Study 1. Participants filled in a short baseline survey comprising socio-demographic questions. Next, the participants read and evaluated Scenario 2, a hypothetical clinical situation that could arise at an emergency department of a hospital (see SI1). Participants were subsequently presented with four different supervision styles of a consultant in response to this particular scenario. To control for order effects, the supervision styles were randomly presented for each participant. After reading about each supervision style, participants completed measures of need frustration and need satisfaction (randomised order), followed by intrinsic motivation. Finally, the participants were asked to rate their preferred supervision style. The *high* need support, *low* directiveness style was preferred by 33 participants (71.7%); 11 participants preferred the *high* need support, *high* directiveness style (23.9%); only two participants preferred the *low* need support, *low* directiveness style (4.3%); and no participants preferred the *low* need support, *high* directiveness style. As participation in Study 2 took substantially more time than Study 1, all participants who completed the survey of Study 2 were invited to share their (work) address to receive a chocolate bar as compensation for their time and effort. Ethical approval for Study 2 was obtained from The Netherlands Association of Medical Education (NVMO, file #2021.8.3).

Participants were recruited from multiple hospitals in the Netherlands between November 2021 and April 2022. We specifically recruited junior doctors who were not in training or were in the first three years of PGME training. We received 57 submissions, of which 46 (80.7%) were complete. We recruited fewer participants than in Study 1, because Study 2 included repeated measurements of each individual participant. Incomplete submissions were removed from the dataset. Testing for outliers in the dependent variables revealed 17 submissions with extreme scores (SD >|3|) in one or more of the dependent variables in some of the experimental conditions. None of these submissions showed generally strongly deviating response patterns. Therefore, they were not excluded from further analysis. The final sample comprised of 46 participants (*n* = 33, 71.7% female), who worked as junior doctors not-in-training (*n* = 24, 52.2%) or as PGME trainees (*n* = 22, 47.8%). More specifically, nine junior doctors (19.6%) were in their first PGME year, six junior doctors (19.6%) were in their second PGME year, six junior doctors (13.0%) were in their third PGME year, and one junior doctor (2.2%) was in their fourth PGME year. Participants’ ages ranged from 23 to 35 years (*M* = 29.00, *SD* = 2.60), they worked in 17 different specialities, and trainees were being trained in 13 different PGME programmes.

#### Measures

The measures used in Study 2 were the same as in Study 1.

#### Statistical analyses

We used the same software packages as in Study 1. Where possible, we replicated the analyses of Study 1, taking into consideration that Study 2 included dependent measurements, i.e., for each experimental condition. Due to the smaller sample size in Study 2, we could not test for unique effects of all predictors in parallel mediation models. Therefore, we modelled the indirect effects of the summarised values of ‘need frustration’ (i.e., M_1_ + M_2_ + M_3_) and ‘need satisfaction’ (i.e., M_4_ + M_5_ + M_6_) as our mediators in both studies.

## Results

Table 4 shows the means, standard deviations, and correlations of the continuous variables across the four supervision styles. All correlations were in the expected direction. Age, sex, and training level again had no meaningful relation with the variables in our research model (Fig. 2).

**Table 4:** Cronbach’s alpha’s, means, standard deviations across the four supervision styles, and correlations (study 2)

Variables	Cronbach’s α	*M*_*Study2*_	*SD*_*Study2*_	2	3	4	5	6	7
1. Autonomy frustration	.75–.94	2.23	1.07	.37–.65	.41–.51	−.53– −.19	−.61– −.22	−.24–.09	−.52– −.21
2. Competence frustration	.85–.95	2.38	1.19	–	.44–.71	−.56– −.17	−.77– −.49	−.32– −.20	−.55– −.34
3. Relatedness frustration	.87–.90	2.08	1.07		–	−.39– −.16	−.52– −.34	−.27– −.10	−.49– −.29
4. Autonomy satisfaction	.67–.91	3.31	1.09			–	.36–.66	.27–.51	.33–.61
5. Competence satisfaction	.83–.93	3.36	1.15				–	.34–.52	.47–.68
6. Relatedness satisfaction	.85–.97	3.06	1.09					–	.26–.52
7. Intrinsic motivation	.90–.94	3.59	1.06						–

*Note. N*_Study2_ = 46. Correlations >|.29| are significant at the *p* <.05 level, correlations >|.45| are significant at the *p* <.001 level. Cronbach’s alphas and correlations are presented as ranges because of the four conditions that were evaluated

### Hypothesis testing

In *Hypothesis 1*, we posited that *compared with high need support, low need support leads to more psychological need frustration and less psychological need satisfaction*. As in Study 1, we performed a 2 × 2 MANOVA, but now for repeated measures. As in Study 1, the results revealed a strong multivariate main effect of need support (see Table 5). Compared with the *high* need-support conditions, participants in the *low* need-support conditions reported significantly more need frustration (*ps* <.001; see Table SI2) and less need satisfaction (*ps* <.001). Hence, also in Study 2, our findings provided empirical support for *Hypothesis 1*.

**Table 5:** 2 × 2 repeated measures MANOVA results with need support (*high* versus* low*) and directiveness (*high* versus* low*) as within-subject factors (Study 2)

	Multivariate *F*(7, 39)	$\eta _p^2$			Univariate *F*(1, 45)	$\eta _p^2$
Need support	39.45***	.88	1.	Autonomy frustration	140.43***	.76
			2.	Competence frustration	143.17***	.76
			3.	Relatedness frustration	123.30***	.73
			4.	Autonomy satisfaction	95.09***	.68
			5.	Competence satisfaction	179.14***	.80
			6.	Relatedness satisfaction	101.42***	.69
			7.	Intrinsic motivation	231.74***	.84
Directiveness	32.36***	.85	1.	Autonomy frustration	124.25***	.73
			2.	Competence frustration	34.49***	.43
			3.	Relatedness frustration	53.28***	.54
			4.	Autonomy satisfaction	174.47***	.80
			5.	Competence satisfaction	129.30***	.74
			6.	Relatedness satisfaction	31.47***	.41
			7.	Intrinsic motivation	91.91***	.67
Need support x Directiveness	16.24***	.75	1.	Autonomy frustration	46.50***	.51
			2.	Competence frustration	46.58***	.51
			3.	Relatedness frustration	30.18***	.40
			4.	Autonomy satisfaction	52.61***	.54
			5.	Competence satisfaction	59.20***	.57
			6.	Relatedness satisfaction	11.16**	.20
			7.	Intrinsic motivation	47.46***	.51

*Note* *p <.05. **p <.01. ***p <.001

In *Hypothesis 2*, we posited that *compared with high need support, low need support leads to more psychological need frustration and less psychological need satisfaction, particularly in the case of high directiveness*. We found empirical support for this hypothesis in Study 2. Table 5 shows the significant multivariate interaction effect of need support and directiveness. Thus, we replicated the interaction effect on competence frustration (see Study 1, Fig. 3), and observed a similar pattern for all the other dependent variables. Specifically, as shown in Table 6, follow-up tests showed that *low* need support, *high* directiveness resulted in the highest scores on psychological need frustration (1–3), and lowest scores on need satisfaction (4–6). These findings provide strong empirical support for *Hypothesis 2*.

**Table 6:** Means and standard deviations per supervision style (Study 2)

	High need support		Low need support	
	Low directiveness	High directiveness	Low directiveness	High directiveness
Variables	*M (SD)*	*M (SD)*	*M (SD)*	*M (SD)*
1. Autonomy frustration	1.59 (0.67)_a_	1.97 (0.92)_b_	1.84 (0.64)_ab_	3.52 (0.77)_c_
2. Competence frustration	1.76 (0.76)_a_	1.70 (0.79)_a_	2.36 (0.87)_b_	3.72 (1.04)_c_
3. Relatedness frustration	1.35 (0.51)_a_	1.53 (0.67)_a_	2.14 (0.85)_b_	3.29 (0.95)_c_
4. Autonomy satisfaction	4.07 (0.76)_a_	3.58 (0.81)_b_	3.70 (0.62)_b_	1.88 (0.57)_c_
5. Competence satisfaction	4.21 (0.52)_a_	4.02 (0.70)_a_	3.36 (0.74)_b_	1.87 (0.77)_c_
6. Relatedness satisfaction	3.79 (0.88)_a_	3.63 (1.00)_a_	2.80 (0.71)_b_	2.02 (0.70)_c_
7. Intrinsic motivation	4.46 (0.58)_a_	4.15 (0.51)_b_	3.54 (0.61)_c_	2.21 (0.73)_d_

*Note* Within each row, means with different subscripts differ by *p* <.05 at the minimum

In *Hypothesis 3a*, we posited that *compared with high need support, low need support leads to less intrinsic motivation through more psychological need frustration and less psychological need satisfaction*. Similar to Study 1, we found a multivariate main effect of need support on intrinsic motivation (see Table 5). Compared with the *high* need-support conditions, participants in the *low* need-support conditions reported significantly less intrinsic motivation (*p* <.001). As expected, Table 7 illustrates that Study 2 replicates the findings of Study 1. The effect of *high* need support on intrinsic motivation was negatively mediated through autonomy, competence, and relatedness frustration, and positively mediated through autonomy, competence, and relatedness satisfaction. The effect of *low* need support on intrinsic motivation was negatively mediated through autonomy, competence, and relatedness frustration, and positively mediated through autonomy and competence satisfaction. Relatedness satisfaction did not significantly mediate the effect of *low* need support on intrinsic motivation. Thus, as in Study 1, we found empirical support for *Hypothesis 3a* in Study 2.

**Table 7:** Indirect effects of high and low need-supportive supervision styles on intrinsic motivation through psychological need frustration and psychological need satisfaction (Study 2)

	High need support						Low need support					
	*ab*	*SE*	95% CI		*p*	Sobel	*ab*	*SE*	95% CI		*p*	Sobel
Indirect effects			*LL*	*UL*					*LL*	*UL*		
Separate models for each variable*												
1. Autonomy frustration	-0.49	0.17	-0.81	-0.14	.004	-0.11	-1.08	0.36	-1.67	-0.21	.002	-0.37
2. Competence frustration	-0.38	0.26	-1.01	-0.07	.143	-0.09	-1.06	0.28	-1.60	-0.51	<.001	-0.37
3. Relatedness frustration	-0.51	0.19	-0.81	-0.05	.007	-0.12	-1.05	0.25	-1.50	-0.51	<.001	-0.37
4. Autonomy satisfaction	1.35	0.36	0.62	2.02	<.001	0.31	1.62	0.49	0.63	2.61	<.001	0.56
5. Competence satisfaction	1.50	0.59	0.48	2.69	.011	0.35	1.54	0.23	1.09	1.97	<.001	0.53
6. Relatedness satisfaction	1.18	0.32	0.61	1.91	<.001	0.27	0.54	0.42	-0.21	1.47	.198	0.19
Summarised models for need frustration and need satisfaction**												
Need frustration	-0.65	0.24	-1.13	-0.20	.007	-0.15	-1.52	0.33	-2.12	-0.79	<.001	-0.53
Need satisfaction	1.93	0.39	1.19	2.75	<.001	0.45	1.87	0.41	1.01	2.61	<.001	0.65

*Note* *p <.008 is considered significant (Bonferroni correction with a factor 6 due to multiple model testing for both satisfaction and frustration). **p <.05 is considered significant. Furthermore, the bootstrapped 95% Confidence Intervals (95% CI) of the indirect effects are considered significant when the parameters of the Lower Limit (*LL*) and Upper Limit (*UL*) do not include zero. All indirect effects (*ab*) are unstandardized. Both standard error (*SE*) and 95% CI, and Sobel’s statistic ((*ab*) / (*ab* + *c’*)), are 1000 bootstrapped estimates. Direct effects are not presented in this table, because the direct effect (*c’*) is the value of the total effect (*c*), which is the mean value of intrinsic motivation for *high* versus *low* need-supportive styles, minus the indirect effect (*ab*)

In *Hypothesis 3b*, we posited that *compared with high need support, low need support leads to less intrinsic motivation through more need frustration and less need satisfaction, particularly in the case of high directive supervision.* As shown in Table 5, we found a multivariate interaction effect of need support and directiveness on intrinsic motivation. Participants in the *low* need-support, *high* directiveness condition reported the lowest scores on intrinsic motivation (see Table 6). Table 8 illustrates that the effect of *low* need support, *high* directiveness on intrinsic motivation was negatively mediated through autonomy, competence, and relatedness frustration, and positively mediated through autonomy and competence satisfaction. However, relatedness satisfaction did not significantly mediate this effect. Thus, in contrast to Study 1, we found partial support for *Hypothesis 3b* in Study 2.

**Table 8:** Indirect effects of the low need-supportive, high directive supervision style on intrinsic motivation through psychological need frustration and psychological need satisfaction (Study 2)

	Low need support, high directiveness						
Indirect effects	Path	est.	*SE*	95% CI		*p*	Sobel
				*LL*	*UL*		
Separate models for each variable*							
1. Autonomy frustration	*ab*	-1.39	0.49	-2.14	-0.17	.005	-0.63
2. Competence frustration	*ab*	-0.88	0.40	-1.77	-0.19	.029	-0.40
3. Relatedness frustration	*ab*	-1.13	0.35	-1.74	-0.28	.001	-0.51
4. Autonomy satisfaction	*ab*	1.36	0.38	0.56	2.07	<.001	0.62
5. Competence satisfaction	*ab*	0.98	0.23	0.52	1.41	<.001	0.44
6. Relatedness satisfaction	*ab*	0.54	0.35	-0.16	1.19	.116	0.24
Summarised models for need frustration and need satisfaction**							
Need frustration	*ab*	-1.77	0.47	-2.57	-0.76	<.001	-0.80
Need satisfaction	*ab*	1.56	0.36	0.84	2.25	<.001	0.71

*Note* *p <.008 is considered significant (Bonferroni correction with a factor 6 due to multiple model testing for both satisfaction and frustration). **p <.05 is considered significant. All indirect effects (*ab*) are unstandardized. Furthermore, the bootstrapped 95% Confidence Intervals (95% CI) of the indirect effects are considered significant when the parameters of the Lower Limit (*LL*) and Upper Limit (*UL*) do not include zero. Both standard error (*SE*) and 95% CI, and Sobel’s statistic ((*ab*) / (*ab* + *c’*)), are 1000 bootstrapped estimates. Direct effects are not presented in this table, because the direct effect (*c’*) is the value of the total effect (*c*), which is the mean value of intrinsic motivation, minus the indirect effect (*ab*)

## Discussion

In this experimental vignette study, we relied on a *between*-subjects (Study 1) and *within*-subjects design (Study 2) to examine the effects of supervision styles on psychological need frustration, psychological need satisfaction, and, accordingly, junior doctors’ intrinsic motivation. The results of both studies consistently showed that compared with *high* need-supportive supervision styles, *low* need-supportive supervision styles hamper intrinsic motivation. The effect of supervision styles on intrinsic motivation was consistently mediated through need frustration and need satisfaction. This research replicates and strengthens previous findings showing the importance of need-supportive supervision styles for postgraduate clinical training.

The effects of *high* versus *low* need-supportive supervision styles on junior doctors’ intrinsic motivation correspond with SDT’s central proposition that socio-contextual conditions may facilitate or hamper motivation (Ryan & Deci, 2017). Need satisfaction, especially autonomy and competence satisfaction, positively mediated the effects of supervision styles on junior doctors’ intrinsic motivation. Similarly, need frustration negatively mediated the effects of supervision styles on junior doctors’ intrinsic motivation. That is, we found empirical support for the dual-process model: namely, that *high* need-supportive styles promote intrinsic motivation through lower levels of need frustration and higher levels of need satisfaction (bright pathway) and *low* need-supportive styles hamper intrinsic motivation through higher levels of need frustration and lower levels of need satisfaction (dark pathway) (Haerens et al., 2015; Ryan & Deci, 2000a; Vansteenkiste & Ryan, 2013).

The current findings show, in addition, that especially the need for autonomy may suffer from directiveness, even when need support is *high*. Indeed, Cognitive Evaluation Theory (CET), another mini-theory of SDT (Vansteenkiste et al., 2010), proposes that a directive style may yield different effects on psychological need satisfaction. When perceived as informational, a directive style may support recipients’ need for competence, and frustrate their need for autonomy. When perceived as controlling, a directive style tends to thwart both competence and autonomy (Vansteenkiste et al., 2010).

Unexpectedly, junior doctors’ age, sex, and training stage showed no meaningful relation with psychological need frustration, psychological need satisfaction, or intrinsic motivation. Based on previous findings in postgraduate training (Olmos-Vega et al., 2015; Sheu et al., 2017), we expected that younger and inexperienced junior doctors might prefer supervision styles that are both need-supportive and directive in situations that exceed their capacities. An explanation for our findings may be that most junior doctors in our samples already had (some) work experience in the emergency department setting. In the Netherlands, many junior doctors work as physicians before they enter postgraduate training. Thus, the postgraduate training year may not (always) correspond with clinical work experience. The same goes for age, further compounded by dispersed age at graduation and enrolment into postgraduate training (Pols et al., 2021).

### Theoretical considerations

Some theoretical considerations with regard to (de)motivating styles need to be addressed. In our research, the *high* need-supportive styles consistently showed the highest levels of psychological need satisfaction and intrinsic motivation. This suggests that both styles were perceived as learner-focused (Reeve & Cheon, 2021) and complementary (Jang et al., 2010). Note however, that the manipulation checks indicated that the *high* need-supportive, *low* directive style was perceived as moderately directive. This suggests that our operationalisation of *high* need-supportive, *low* directive supervision may have been perceived as an attuning approach, which closely relates to *high* need-supportive, *high* directive styles (Aelterman et al., 2018).

Both low need-supportive styles revealed detrimental outcomes compared with the *high* need-supportive styles. The expected negative effect of *low* need-supportive, *high* directive supervision was only evident in Study 2, possibly because junior doctors evaluated this style more negatively compared with the other styles (Aguinis & Bradley, 2014; Atzmüller & Steiner, 2010). Surprisingly, in Study 2, the *low* need-supportive, *low* directive style was perceived as less demotivating than the *low* need-supportive, *high* directive style. This contrasts with the previous literature, which shows that the *low* need-supportive, *low* directive style is perceived as equally detrimental as the *low* need-supportive, *high* directive style (Aelterman et al., 2018; Delrue et al., 2019). However, it has also been found that sport coaches and teachers who are low in autonomy support and control yield motivational outcomes that are less negative than high controlling sport coaches and teachers (Haerens et al., 2018). Regardless, it is also possible that the *low* need-supportive, *low* directive style in Study 2 was perceived as an awaiting approach instead of an abandoning approach (Aelterman et al., 2018). As a result, some junior doctors may perceive an awaiting style as an invitation to discover opportunities on their own. This might be beneficial for junior doctors who feel already quite confident in their own abilities, but are hesitant to take the leap towards independent practice. For inexperienced junior doctors, however, an awaiting style may result in insecurity, especially when they feel there is no safety net to safeguard patient care and debriefing afterwards to discuss supervisors’ considerations. Drawing on the crucial importance of need satisfaction within SDT, we pose that repeated experiences of *low* need-supportive, *low* directive supervision styles come with psychological costs that, besides their impact on intrinsic motivation, will hamper future learning and development. Future research is needed to examine whether the operationalisation of (de)motivating styles requires nuances in postgraduate training that differ from class-based education, where most research about (de)motivating styles has been conducted (Vansteenkiste et al., 2019).

Some effects of (de)motivating styles may be explained through the specific design of our study. Our scenarios manipulated the (de)motivating styles of fictitious consultants in specific situations, but did not provide an evaluation of the supervisory practices of actual consultants. This differs from previous (vignette) studies that focused on the general (de)motivating styles of actual coaches (Delrue et al., 2019; Haerens et al., 2018), teachers (Aelterman et al., 2018; Jang et al., 2010; Neufeld & Malin, 2020), or nurses (Duprez et al., 2019). It is possible that general motivational styles may have stronger or different effects on need-based experiences and motivation than (de)motivating styles in specific situations; this was also suggested in previous research with physical education teachers (Haerens et al., 2018). Thus, the effects of (de)motivating styles are not directly comparable between studies.

### Practical implications

Our research findings show that need-supportive supervision styles play an important role in motivating junior doctors in postgraduate clinical training settings. Building upon findings of previous studies (e.g., Apramian et al., 2015, 2016; Goldszmidt et al., 2015), the present study provides theoretical underpinnings of the effects of supervision styles in PGME on junior doctors’ psychological needs and motivation. In the longer term, supervision styles are likely to have an impact on junior doctors’ mind-sets, (future) behaviour regulation, and learning, regardless of the intentions of their supervisors. Hence, we specifically recommend that consultants adopt need-supportive supervision styles to promote junior doctors’ intrinsic motivation through psychological need satisfaction. We recommend first to invest in the need for relatedness to build mutual trust (Hauer et al., 2014). Familiarity and regular contact between supervisor and junior doctor, and support in a dynamic clinical practice, are essential elements for building a trusting relationship. This is important because providing patient care will always remain a balancing act between patient safety and learning in practice (Hoffman, 2015). Thus, consultants need to act as role models and create a safe environment where junior doctors can learn and fail with limited risks to patients.

Second, we suggest that short briefing and debriefing sessions (e.g., around shifts) can also facilitate psychological need support. For example, before shifts, supervisors and junior doctors can discuss expectations and set boundaries for supervision. At the end of shifts, a short evaluation of the collaboration can help in reflecting on learning goals and critical incidents. Such actions will likely satisfy the need for competence by establishing what the junior doctor already knows and to what extent, and how the consultant can adapt supervision to the specific learning goals of the junior doctor. The need for autonomy will likely be supported when supervisors and junior doctors determine where there is room for volitional functioning and choice, and how junior doctors’ active involvement in learning can be supported. During these (de)briefing sessions, the learners’ perspectives and learning goals should be central (Vansteenkiste et al., 2019). This requires customisation and calibration of supervision for individual junior doctors. This is important because they are being trained as future consultants and need to learn the ropes.

Previous studies suggest that people (i.e., consultants) can be trained to use styles and strategies that are need-supportive rather than need thwarting and controlling (e.g., Hardré & Reeve, 2009; Neufeld, 2021; Reeve & Cheon, 2021; Vansteenkiste et al., 2019; Vansteenkiste et al., 2020). Faculty development programmes are likely to be more effective when they are tailored to consultants’ considerations, preferences, and beliefs that underly their supervisory practices (Apramian et al., 2016; Goldszmidt et al., 2015). These need to be addressed because supervisory practices vary (Kennedy et al., 2007), differences in supervision styles are not regularly discussed in practice (Apramian et al., 2016; Goldszmidt et al., 2015), and supervisors and junior doctors may evaluate supervision differently (Biondi et al., 2015). Thus, to train consultants in using need-supportive supervision styles, we suggest developing an individualised faculty development training programme. The scenarios developed in the present studies can be used for such a training programme which might consist of six phases. First, we suggest reflecting on different scenarios to develop an understanding of individual supervisory preferences and practices, and their potential impact on junior doctors. Second, some theoretical background concerning basic psychological needs and motivational strategies needs to be provided to create a shared understanding of need-supportive supervision. Third, the scenarios can be used as inspiration for how to apply need-supportive supervision clinical practice. Consultants are invited to share, discuss, and reflect on examples from their practice settings and experiences from their own training. Need-supportive experiences may differ between individuals and settings. Fourth, practical exercises, e.g., role-plays, can help in developing need-supportive supervision styles and receiving targeted feedback on these. Fifth, video recordings of actual supervision can be used to reflect on and better tailor need-supportive styles in practice. Finally, because supervisory styles and practices vary, it can be helpful to create guided intervision (i.e., peer coaching) groups to regularly discuss challenges in clinical supervision. Training and reflecting on need-supportive supervision styles is relevant for both postgraduate training and other HPE programmes (e.g., nursing, physiotherapy) to better support learners’ intrinsic motivation in clinical practice.

### Strengths and limitations

A strength of this research is that our *between*- and *within*-subjects experimental designs allow for causal interference and mediation analysis. Hence, the present research adds to the growing body of literature aimed at providing a better understanding of motivational pathways. Furthermore, vignette designs permit examination of (de)motivating supervision styles without the potential harm to junior doctors of exposing them to these styles in practice; they also reduce the confounding factors that may arise in clinical practice due to its contextual dynamics (Berkhout et al., 2018; van der Goot et al., 2020). The ecological validity of both studies was enhanced by our reliance on samples of actual junior doctors who receive supervision from consultants daily. In addition, we used validated scales, and to control for order effects, presented the items within each scale in random order (Carpenter et al., 1993; Chen et al., 2015; Van Yperen, 1998).

The simultaneous presentation of four different vignettes with one supervision style in Study 1 may have resulted in a better-informed judgment of that specific supervision style. In Study 2, we exposed the four supervision styles in random order to each participant. As a result, in Study 2, the participants had the opportunity to build a better-informed judgment about the different supervision styles. This may explain respondents’ clearly stated preferences for a need-supportive supervision style at the end of Study 2. Important to note, however, is that the findings of both studies were largely comparable. Nuanced differences in outcomes between the two designs (i.e., judgment of one style in four situations in Study 1 versus comparison between four styles in one situation in Study 2) was most clearly shown for the *low* need-supportive styles. Specifically, the detrimental effect of a *low* need-supportive, *high* directive style was only partially found in Study 1, but became pronounced when junior doctors could compare all four styles. A potential reason may be that junior doctors could imagine and feel their inner responses to different styles more clearly when they compared all four styles. Moreover, a *low* directive style allows junior doctors to ask other healthcare professionals for help– when needed, while a *high* directive styles clearly prescribes what the junior doctor must do.

Our research also has limitations. First, *between-* and *within-*subjects designs have their limitations (Charness et al., 2012). *Between-*subjects designs need larger sample sizes to obtain sufficient statistical power. Although our sample size was large enough to test our hypotheses, the different conditions were not perfectly balanced due to participants withdrawing during data collection. It is possible that some effects of individual characteristics of junior doctors (e.g., work experience, speciality choice, and sex) were not found as a result. In our *within*-subjects design, however, we could not rely on conventional manipulation checks, as was done in Study 1. Although, as we have demonstrated in Study 1, our manipulations were successful, we cannot be sure whether the participants perceived the intended differences between the supervision styles in Study 2.

Second, the design of this research, with experimentally manipulated, categorical independent variables, complicated the mediation analyses and limited the options for calculating the effect sizes of the indirect effects. As a result, only the direct effect (*b-*path) between our mediating variables and intrinsic motivation could be modelled. The direct effect between supervision style and mediator (*a*-path) and the total effect of supervision style on intrinsic motivation (*c*-path) were simply the mean scores of these variables. Moreover, the sample size of Study 2 was too small to test a full mediation model. Therefore, in Study 2, we could not identify the unique contributions of the individual basic psychological needs. Although we are quite confident about our consistent findings across both studies, future research needs larger sample sizes to fully apply mediation analysis.

Third, although we drew from the circumplex approach to (de)motivating styles (Aelterman et al., 2018; Delrue et al., 2019; Duprez et al., 2019), for reasons of parsimony and feasibility, we focused on four rather than eight different styles. Hence, we ignored that, in clinical practice, supervision styles can cover a whole range of approaches. Many different interactions take place between junior doctors and consultants, but also between junior doctors and other health professionals. Future studies may examine multiple scenarios with different supervision styles, or use additional cues, such as tone of voice or non-verbal behaviour, to more realistically represent different (de)motivating styles. In addition, observational studies may help to better identify supervision styles in different clinical settings and the interpersonal dynamics between junior doctors and consultants. Regardless, the positive effects of need-supportive supervision styles found in this study are in line with previous research findings in other domains. Thus, we expect that need-supportive supervision will enhance the intrinsic motivation, and healthy development, of junior doctors even when situations and contexts change.

Finally, although we investigated basic psychological need frustration and satisfaction using a measure specifically developed for adult populations and that was validated in Dutch (Chen et al., 2015; Vansteenkiste et al., 2020), it is possible that domain-specific measures would better capture need-based experiences in postgraduate clinical training. One reason is that this setting may provide unique challenges and characteristics that need to be captured, such as relative unpredictability due to the mix of patients and the shared responsibility for patient care between junior doctors and consultants. Furthermore, we only measured intrinsic motivation in this research and, therefore, did not fully assess the negative motivational pathway (Bartholomew et al., 2011; Haerens et al., 2015). Particularly, because junior doctors have busy and demanding jobs, which can affect the recruitment of participants, we kept the surveys as short as possible. However, it is likely that different supervision styles affect the whole spectrum from intrinsic to extrinsic motivation, and even amotivation. Therefore, future research might include a broader range of motivational measures to fully assess the effects of psychological need frustration and psychological need satisfaction.

## Conclusions

*High* need-supportive supervision styles are important to promote intrinsic motivation through need satisfaction in junior doctors. Researchers and (clinical) educators in HPE could use SDT as a framework to further unravel clinical supervision, and develop interventions to enhance need-supportive approaches to supervision.

## Electronic supplementary material

Below is the link to the electronic supplementary material.

Supplementary Material 1

Supplementary Material 2

## Data Availability

Due to regulations and restrictions related to the confidentiality of participants, data are not publicly available. Requests for data can be addressed to the programme leader of LEARN (learn@umcg.nl). Requests will be evaluated on a case-by-case basis. Researchers seeking to replicate our findings can request a copy of the dissertation in which the methodological considerations are presented and discussed in more detail (Chap. 4): 10.33612/diss.936613878.

## Footnotes

1 In our information letter we stated that participants could withdraw from the study by not completing the survey.

2 The percentage of female medical students (undergraduate) in the Netherlands ranges between 66.5% and 68.4% (2016–2022, CBS Statline, https://opendata.cbs.nl/statline/#/CBS/nl/dataset/83538NED/table?dl=758B1).
