# Supplementary material for: Effects of (de)motivating supervision styles on junior doctors’ intrinsic motivation through basic psychological need frustration and satisfaction: an experimental vignette study
Source: Adv Health Sci Educ Theory Pract. 2024 Jun 25;30(2):401–26. doi: 10.1007/s10459-024-10344-0 (PMC11965158; doi:10.1007/s10459-024-10344-0)
Supplement: Supplementary file 1 — Supplementary Material 1 [file 10459_2024_10344_MOESM1_ESM.docx]

# **Effects of (de)motivating supervision styles on junior doctors’ intrinsic motivation through basic psychological need frustration and satisfaction:**

**An experimental vignette study**

**Advances in Health Sciences Education**

Wieke E. van der Goot^1,2^, Nico W. Van Yperen^3^, Casper J. Albers^3^, A. Debbie C. Jaarsma^2,4^, Robbert J. Duvivier^2,5^

^1^Martini Academy, Martini Hospital, Groningen, the Netherlands

^2^University of Groningen, University Medical Center Groningen, Lifelong Learning, Education and Assessment Research Network (LEARN), Groningen, the Netherlands

^3^University of Groningen, Department of Psychology, Groningen, the Netherlands

^4^Faculty of Veterinary Medicine, University of Utrecht, Utrecht, the Netherlands

^5^Parnassia Psychiatric Institute, The Hague, the Netherlands

Corresponding author: [w.e.van.der.goot@rug.nl](mailto:w.e.van.der.goot@rug.nl)

**Supplementary Information 1: Pilot Study**

**Scenario development and pilot testing**

The scenarios were developed in 2019. We described situations that junior doctors may encounter when they work at the emergency department. Four junior doctors, from both surgical and non-surgical specialties, read and evaluated the preliminary scenarios and supervision styles to improve their realism and recognisability (ecological validity). We also developed manipulation checks.

In January and February 2020, the scenarios were pilot tested in two cohorts of first-year interns (paper-and-pencil survey). After the first pilot test, we made some minor textual adjustments to some of the supervision styles and manipulation check items to improve their clarity. Participants were informed about the study and provided informed consent before the start of the pilot study. Ethical approval for this study was obtained from The Netherlands Association of Medical Education (NVMO, file #2019.8.3).

Participants randomly received a survey with one of the four experimental conditions, which differed in need support (high versus low) and directiveness (high versus low). The distribution of conditions was balanced in both cohorts. Participants evaluated eight scenarios, and rated the manipulation check items. After the pilot tests, the four scenarios that were deemed most realistic for junior doctors were selected for Study 1 (see Scenarios). We assessed need support and directiveness using five items per factor. Respondents rated these items on a seven-point Likert scale. Finally, there were open-ended questions, giving participants the opportunity to comment on the supervision styles and provide suggestions for improvement.

The first pilot cohort comprised 52 participants (n = 33, 63.5% female), with an average age of 22.6 years (*SD* = 1.60, range 21 – 28); 23 participants (44.2%) had some work experience at the emergency department. The second pilot cohort comprised 49 participants (n = 29, 59.2% female), with an average age of 23.0 years (*SD* = 1.72, range 21 – 29); 28 had some work experience at the emergency department (57.1%). In Cohort 1, 38 participants (73.1 %) identified the correct supervision style; in Cohort 2, 34 participants (69.4 %) did so.

We calculated the Cronbach’s alphas for both scales. The results revealed high reliability of the need-support manipulation checks in both Cohorts: .95 (Cohort 1) and .96 (Cohort 2), respectively. Inter-item correlations revealed that all items were strongly correlated. The three items that had the largest impact on the Cronbach’s alpha of the need-support scale were used in our final manipulation-check scale. The final need-support scale had a Cronbach’s alpha of .90. The results of the directiveness manipulation checks revealed high reliability in both cohorts as well: .82 (Cohort 1) and .83 (Cohort 2), respectively. Inter-item correlations revealed that all items were strongly correlated. The three items with the largest impact on the Cronbach’s alpha of the directiveness scale were included in our final manipulation-check scale. The final directiveness scale had a Cronbach’s alpha of .83.

To test the effectiveness of the manipulation checks, we performed a 2 (need support: high versus low) x 2 (directiveness: high versus low) MANOVA. The results of Cohort 1 revealed a significant main effect of need support, *F*(2, 46) = 67.51, p < .00, η^2^_p_= .75, and directiveness, *F*(2, 46) = 35.94, p < .00, η^2^_p_= .61, but no significant interaction effect, *F*(2,46) = 1.81, *p* =.174, η^2^_p_= .07. The results of Cohort 2 indicated a significant main effect of need support, *F*(2, 44) = 36.53, p < .00, η^2^_p_= .62, and a significant main effect of directiveness, *F*(2, 44) = 23.80, p < .00, η^2^_p_= .52, but no significant interaction effect, *F*(2,44) = 1.67, *p* =.200, η^2^_p_= .07. Both cohorts revealed similar response patterns (see Table SI1). Univariate analyses revealed that participants in the high need-support conditions scored significantly higher on the need-support manipulation checks than the participants in the low need-support conditions. In addition, participants in the high directiveness conditions scored significantly higher on the directiveness manipulation checks than participants in the low directiveness conditions (see, Figures SI1 and Figure SI2; only Cohort 1 is shown in the figures).

**Table SI1**

*Means and Standard Deviations Need Support and Directiveness (Cohorts 1 and 2)*

|  | Experimental condition | | | |
| --- | --- | --- | --- | --- |
|  | Need support | | Directiveness | |
|  | Low | High | Low | High |
| Variables | M (SD) | M (SD) | M (SD) | M (SD) |
| Cohort 1 |  |  |  |  |
| 1. Need support | 2.73* (0.82) | 5.24* (0.72) |  |  |
| 2. Directiveness |  |  | 3.54* (0.83) | 5.46* (0.91) |
| Cohort 2 |  |  |  |  |
| 1. Need support | 2.65* (1.06) | 5.15* (0.98) |  |  |
| 2. Directiveness |  |  | 3.29* (1.09) | 5.21* (0.86) |

*Note.* *Main effects are significant, *p* < .001 at the minimum.

**Figure SI1**

*Average Score Pattern for the Need-Support Manipulation Checks (Cohort 1)*


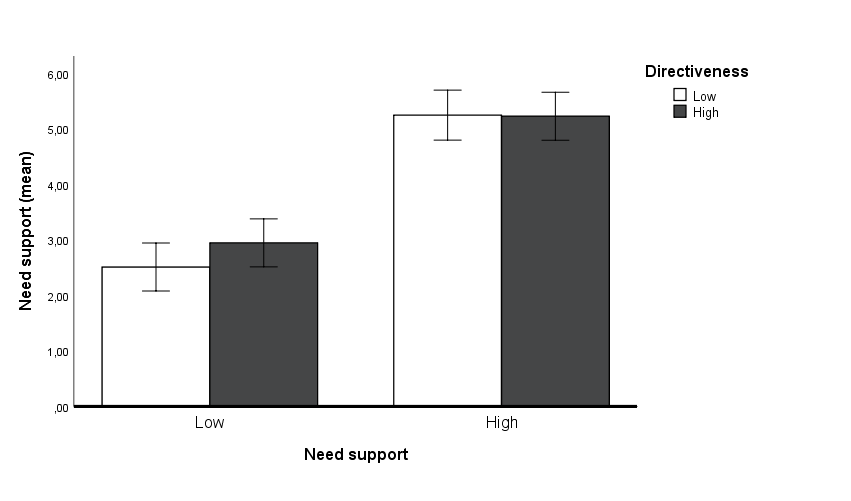


*Note.* The low need-support conditions, compared with the high need-support conditions, revealed significantly lower scores on the need-support manipulation checks.

**Figure SI2**

*Average Score Pattern for the Directiveness Manipulation Checks (Cohort 1)*


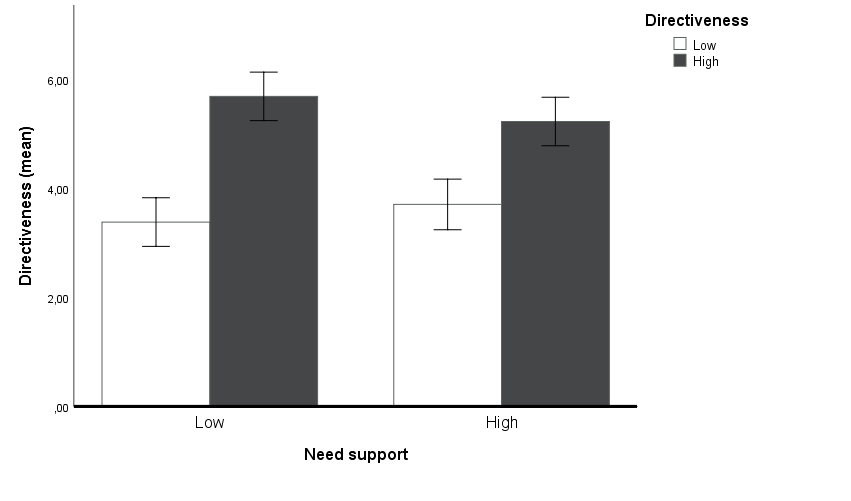


*Note.* The low directiveness conditions, compared with the high directiveness conditions, revealed significantly lower scores on the directiveness manipulation checks.

**Manipulation check items**

Need support

The supervisor from the scenarios…

…is open to my questions (…staat open voor mijn vragen)

…guides me to further develop my clinical skills (…begeleidt me om klinische vaardigheden verder te ontwikkelen)

…supports me in my actions (…ondersteunt me in mijn handelen)

Directiveness

The supervisor from the scenarios…

…gives direction (…geeft nadrukkelijk richting)

…takes the lead (…neemt de leiding)

…is in charge (…heeft de regie in handen)

**Scenarios (English)**

**Scenario 1 (EN)**

You are working at the emergency department today. The ambulance calls and reports a vitally unstable patient with a problem you have not encountered often. You call your supervisor because you want them to come to the emergency department (ED) to take care of this patient together with you.

1. Condition: High need support, low directiveness
   1. The supervisor answers the phone and asks you why you want them to come to the ED at this particular moment. You discuss and the supervisor says, “Fine, I'll be right there. Do you feel you have it under control for now or is there anything else I can do for you?”
2. Condition: High need support, high directiveness
   1. The supervisor picks up the phone and says, “It sounds like you could use some help. I'll be right over.” The supervisor gives you a plan of action over the phone for when the patient arrives.
3. Condition: Low need support, low directiveness
   1. The supervisor responds to your question as follows: “You're a doctor too, aren't you? Go see the patient.”
4. Condition: Low need support, high directiveness
   1. The supervisor picks up the phone and says, “Indeed, you should not do this yourself. Don't do anything until I get there, then I'll show you how to do it.”

**Scenario 2 (EN)**

You are working at the emergency department today. The ambulance calls and reports a stable patient with a problem you have not seen before. You decide to see the patient yourself first. After taking the medical history and doing a physical examination, you have an idea of possible next steps, but you are unsure of what exactly is going on and how to proceed. You call your supervisor for help and advice.

1. Condition: High need support, low directiveness
   1. The supervisor picks up the phone, listens to your story, and asks what you are unsure about. The supervisor encourages you to think for yourself about the best next steps to take in order to arrive at a differential diagnosis and workup.
2. Condition: High need support, high directiveness
   1. The supervisor picks up the phone and asks about your findings and what is not yet clear. The supervisor explains what might be going on and makes a proposal for additional workup. Together you make a plan and your supervisor asks you to call again when the first results of the additional tests are in.
3. Condition: Low need support, low directiveness
   1. The supervisor picks up the phone and during the conversation seems to think everything you say is fine.
4. Condition: Low need support, high directiveness
   1. The supervisor interrupts you at the first hiccup in your story and says, "No, this is not working well. Start again. Halfway through your summary of the physical examination, the supervisor indicates that it is taking too long and says, 'Just use this working diagnosis and request the next additional tests.

**Scenario 3 (EN)**

It is Friday afternoon and you are working at the emergency department. A patient referred by the GP out-of-hours clinic comes in with an unclear, but - it seems - not life-threatening problem. The problem has been going on for some time. The family tells you that they “can't go on like this” and that the patient cannot go into the weekend like this. After anamnesis and physical examination, it is unclear what is going on. You wonder whether the patient should perhaps be admitted after all. You decide to consult with your supervisor.

1. Condition: High need support, low directiveness
   1. You call the supervisor and present the situation, after which the supervisor responds as follows: “What are your doubts and how can I support your thought process?”
2. Condition: High need support, high directiveness
   1. The supervisor goes over your findings and considerations with you in a structured manner and goes over a plan with you on how to go about determining whether this patient should be admitted.
3. Condition: Low need support, low directiveness
   1. You call the supervisor and present the situation. The supervisor responds as follows: “If you think so then you should do it, right?”
4. Condition: Low need support, high directiveness
   1. You call the supervisor and present the situation. The supervisor responds as follows: “Why are you in doubt? What exactly have you done so far? Do additional tests first and call me back when you have a concrete and fully developed plan.”

**Scenario 4 (EN)**

It is night-time. You are on duty in the emergency department (ED) overnight. A hemodynamically unstable patient with poor vital signs comes in. You see the patient and then request additional testing. You expect a life-threatening situation is developing. You call your supervisor because you want them to come.

1. Condition: High need support, low directiveness
   1. The supervisor responds as follows: “I'll be right there. Do you feel like you have it under control?”
2. Condition: High need support, high directiveness
   1. The supervisor responds as follows: “I'm coming right over. Would you also like to request x and y in advance, so we can look at the results and take action. And please call the ICU as well, so they can come to the ER, too.”
3. Condition: Low need support, low directiveness
   1. The supervisor responds as follows: “It won't be that bad. There is nothing wrong now, is there? If it gets worse, I'll be there soon enough.”
4. Condition: Low need support, high directiveness
   1. The supervisor responds as follows: “You want me to come? I want you to call the ICU right now to come to the ED and also request these additional tests. When I get there in a minute, I'll show you what else needs to be done.”

**Scenarios (Dutch)**

**Scenario 1 (Dutch)**

Je werkt vandaag op de spoed. De ambulance belt en meldt een vitaal bedreigde patiënt aan met een probleem dat je nog niet vaak hebt gezien. Je belt je supervisor omdat je wil dat hij/zij naar de spoed komt om samen met jou deze patiënt op te vangen.

1. Conditie: need support hoog, directiveness laag
   1. De supervisor neemt de telefoon op en vraagt je waarom je wilt dat hij/zij nu naar de spoed komt. Jullie overleggen en de supervisor zegt “Prima, ik kom eraan. Heb je het gevoel dat je het voor nu onder controle hebt of kan ik nog wat voor je betekenen?”
2. Conditie: need support hoog, directiveness hoog
   1. De supervisor neemt de telefoon op en zegt: “het klinkt alsof je wel wat hulp kunt gebruiken. Ik kom er zo aan.” De supervisor geeft je alvast telefonisch een plan van aanpak mee voor als de patiënt straks arriveert.
3. Conditie: need support laag, directiveness laag
   1. De supervisor reageert op je vraag als volgt: “Jij bent toch ook dokter? Ga de patiënt maar zien.”
4. Conditie: need support laag, directiveness hoog
   1. De supervisor neemt de telefoon op en zegt: “Dit moet je inderdaad niet zelf doen. Doe nu niets meer totdat ik er ben, dan laat ik zien hoe het moet.”

**Scenario 2 (Dutch)**

Je werkt vandaag op de spoed. De ambulance belt en meldt een niet-vitaal bedreigde patiënt aan met een probleem dat je nog niet eerder hebt gezien. Je besluit de patiënt eerst zelf te zien. Na anamnese en lichamelijk onderzoek heb je wel een idee wat mogelijke vervolgstappen zijn, maar je twijfelt over wat er precies aan de hand is en hoe je verder moet. Je belt je supervisor voor hulp en advies.

1. Conditie: need support hoog, directiveness laag
   1. De supervisor neemt de telefoon op, luistert naar je verhaal en vraagt je waarover je twijfelt. De supervisor stimuleert je om zelf te bedenken welke vervolgstappen je nu het beste kunt zetten om te komen tot een werkdiagnose en beleid.
2. Conditie: need support hoog, directiveness hoog
   1. De supervisor neemt de telefoon op en vraagt je naar je bevindingen en wat nog niet helder is. De supervisor geeft uitleg over wat er aan de hand kan zijn en doet een voorstel voor aanvullend onderzoek. Samen maken jullie een plan en je supervisor vraagt je om weer te bellen als de eerste uitslagen van het aanvullend onderzoek binnen zijn.
3. Conditie: need support laag, directiveness laag
   1. De supervisor neemt de telefoon op en lijkt tijdens het gesprek alles wat je zegt wel prima te vinden
4. Conditie: need support laag, directiveness hoog
   1. De supervisor onderbreekt je bij de eerste hapering in je verhaal en zegt: “Nee, dit werkt zo niet goed. Begin maar even opnieuw. Vertel mij nu eerst eens gestructureerd de voorgeschiedenis, anamnese, lichamelijk onderzoek…” Halverwege je samenvatting van het lichamelijk onderzoek geeft de supervisor aan dat het te lang duurt en zegt “Hanteer nu maar deze werkdiagnose en vraag het volgende aanvullend onderzoek aan.”

**Scenario 3 (Dutch)**

Het is vrijdagmiddag en je werkt op de spoed. Een door de huisartsenpost verwezen patiënt komt binnen met een onduidelijk, maar – naar het lijkt – niet een levensbedreigend probleem. Het probleem speelt al enige tijd. De familie vertelt dat het “zo echt niet meer gaat” en dat de patiënt het weekend zo niet in kan. Na anamnese en lichamelijk onderzoek is het onduidelijk wat er aan de hand is. Je vraagt je af of de patiënt misschien toch opgenomen moet worden. Je besluit met je supervisor te overleggen.

1. Conditie: need support hoog, directiveness laag
   1. Je belt de supervisor en legt de situatie voor waarna de supervisor als volgt reageert: “Waar twijfel je over en hoe kan ik met je meedenken?”
2. Conditie: need support hoog, directiveness hoog
   1. De supervisor neemt gestructureerd je bevindingen en overwegingen met je door en neemt een plan met je door hoe je kunt gaan bepalen of deze patiënt wel of niet opgenomen moet worden.
3. Conditie: need support laag, directiveness laag
   1. Je belt de supervisor en legt de situatie voor waarna de supervisor als volgt reageert: “Als jij dat denkt dan moe je dat maar doen toch?”
4. Conditie: need support laag, directiveness hoog
   1. Je belt de supervisor en legt de situatie voor waarna de supervisor als volgt reageert: “Hoezo twijfel je? Wat heb je precies gedaan tot nu toe? Doe eerst aanvullend onderzoek en bel me terug als je een concreet en uitgewerkt plan hebt.”

**Scenario 4 (Dutch)**

Het is nacht. Je hebt vannacht dienst op de spoed. Er komt een hemodynamisch instabiele patiënt, met slechte vitale functies, binnen. Je ziet de patiënt en hebt vervolgens aanvullend onderzoek aangevraagd. Je verwacht dat er een levensbedreigende situatie gaat ontstaan. Je belt je supervisor omdat je wilt dat hij/zij in huis komt.

1. Conditie: need support hoog, directiveness laag
   1. De supervisor reageert hierop als volgt: “Ik kom er meteen aan. Heb je het gevoel dat je het onder controle hebt?”
2. Conditie: need support hoog, directiveness hoog
   1. De supervisor reageert hierop als volgt: “Ik kom eraan. Wil je ook x en y alvast aanvragen, dan bekijken we zo meteen de uitslagen en ondernemen we actie. En bel de IC ook alvast, dan kan die ook naar de spoed komen.”
3. Conditie: need support laag, directiveness laag
   1. De supervisor reageert hierop als volgt: “Het zal wel meevallen. Er is nu toch niets aan de hand? Als het verslechtert dan ben ik er snel genoeg.”
4. Conditie: need support laag, directiveness hoog

De supervisor reageert hierop als volgt: “Je wilt dat ik in huis kom? Ik wil dat je nu meteen de IC belt om naar de spoed te komen en dit aanvullend onderzoek aanvraagt. Als ik er zo ben laat ik zien wat er nog meer moet gebeuren.”
