# Supplementary material for: Effects of (de)motivating supervision styles on junior doctors’ intrinsic motivation through basic psychological need frustration and satisfaction: an experimental vignette study
Source: Adv Health Sci Educ Theory Pract. 2024 Jun 25;30(2):401–26. doi: 10.1007/s10459-024-10344-0 (PMC11965158; doi:10.1007/s10459-024-10344-0)
Supplement: Supplementary file 2 — Supplementary Material 2 [file 10459_2024_10344_MOESM2_ESM.docx]

# **Effects of (de)motivating supervision styles on junior doctors’ intrinsic motivation through basic psychological need frustration and satisfaction:**

**An experimental vignette study**

**Advances in Health Sciences Education**

Wieke E. van der Goot^1,2^, Nico W. Van Yperen^3^, Casper J. Albers^3^, A. Debbie C. Jaarsma^2,4^, Robbert J. Duvivier^2,5^

^1^Martini Academy, Martini Hospital, Groningen, the Netherlands

^2^University of Groningen, University Medical Center Groningen, Lifelong Learning, Education and Assessment Research Network (LEARN), Groningen, the Netherlands

^3^University of Groningen, Department of Psychology, Groningen, the Netherlands

^4^Faculty of Veterinary Medicine, University of Utrecht, Utrecht, the Netherlands

^5^Parnassia Psychiatric Institute, The Hague, the Netherlands

Corresponding author: [w.e.van.der.goot@rug.nl](mailto:w.e.van.der.goot@rug.nl)

**Table SI 2**

*Means and Standard Deviations of all Dependent Variables for* High *and* Low *Need Support (Study 1 and Study 2)*

|  | Study 1 | |  | Study 2 | |
| --- | --- | --- | --- | --- | --- |
|  | Need support | |  | Need support | |
|  | High | Low |  | High | Low |
| Variables | *M (SD)* | *M (SD)* |  | *M (SD)* | *M (SD)* |
| 1. Autonomy frustration | 1.53 (0.66) | 2.84 (0.80) |  | 1.78 (0.82) | 2.68 (1.10) |
| 2. Competence frustration | 1.43 (0.66) | 2.89 (1.07) |  | 1.73 (0.77) | 3.04 (1.17) |
| 3. Relatedness frustration | 1.23 (0.54) | 2.61 (1.00) |  | 1.44 (0.60) | 2.72 (1.07) |
| 4. Autonomy satisfaction | 3.71 (0.73) | 2.69 (0.77) |  | 3.82 (0.82) | 2.79 (1.09) |
| 5. Competence satisfaction | 4.35 (0.66) | 2.65 (0.87) |  | 4.11 (0.62) | 2.61 (1.06) |
| 6. Relatedness satisfaction | 3.65 (0.73) | 2.52 (0.69) |  | 3.71 (0.94) | 2.41 (0.81) |
| 7. Intrinsic motivation | 4.36 (0.71) | 2.63 (0.92) |  | 4.30 (0.56) | 2.88 (0.95) |

*Note.* For each variable in Study 1 or Study 2, the means between *high* and *low* need support differ by *p* < .001.

**Table SI 3**

*Indirect Effects of Need-Supportive Supervision and Intrinsic Motivation Through Psychological Need Frustration and Psychological Need Satisfaction (Parallel Mediation Model, Study 1)*

|  | High need support | | | | | | | |  | Low need support | | | | | | |
| --- | --- | --- | --- | --- | --- | --- | --- | --- | --- | --- | --- | --- | --- | --- | --- | --- |
|  | Path | *b* | *SE* | 95% CI | | *p* | | Sobel |  | *b* | *SE* | 95% CI | | *p* | | Sobel |
| Indirect effects |  |  |  | *LL* | *UL* |  | |  |  |  |  | *LL* | *UL* |  | |  |
|  |  |  |  |  |  |  |  |  |  |  |  |  |  |  |  |  |
|  | *c’* | 2.24 | 0.82 | 1.10 | 4.22 | .007 | |  |  | 2.24 | 0.77 | 0.79 | 3.91 | .004 | |  |
| 1. Autonomy frustration | *a_1_b_1_* | -0.25 | 0.19 | -0.57 | 0.18 | .176 | | -0.13 |  | -0.23 | 0.35 | -0.92 | 0.48 | .507 | | -0.12 |
| 2. Competence frustration | *a_2_b_2_* | -0.26 | 0.24 | -0.76 | 0.17 | .278 | | -0.13 |  | -0.26 | 0.32 | -0.89 | 0.36 | .410 | | -0.13 |
| 3. Relatedness frustration | *a_3_b_3_* | 0.05 | 0.18 | -0.32 | 0.36 | .790 | | 0.02 |  | -0.44 | 0.29 | -1.06 | 0.07 | .128 | | -0.24 |
| 4. Autonomy satisfaction | *a_4_b_4_* | 0.84 | 0.42 | 0.04 | 1.61 | .045 | | 0.27 |  | 0.62 | 0.38 | -0.18 | 1.36 | .106 | | 0.22 |
| 5. Competence satisfaction | *a_5_b_5_* | 1.62 | 0.71 | -0.09 | 2.53 | .021 | | 0.42 |  | 0.70 | 0.39 | -0.06 | 1.44 | .074 | | 0.24 |
| 6. Relatedness satisfaction | *a_6_b_6_* | 0.12 | 0.30 | -0.46 | 0.69 | .682 | | 0.05 |  | 0.00 | 0.42 | -0.87 | 0.75 | .992 | | 0.00 |

*Note.* *p* < .05 is considered significant. Furthermore, the bootstrapped 95% Confidence Intervals (95% CI) of the indirect effects are considered significant when the parameters of the Lower Limit (*LL*) and Upper Limit (*UL*) do not include zero. All indirect effects are unstandardized. Both standard error (*SE*) and 95% CI, and Sobel’s statistic ((*ab*) / (*ab* + *c’*)), are 1000 bootstrapped estimates.
